# Evaluation of Major Constituents of Medicinally Important Plants for Anti-Inflammatory, Antidiabetic and AGEs Inhibiting Properties: In Vitro and Simulatory Evidence

**DOI:** 10.3390/molecules27196715

**Published:** 2022-10-09

**Authors:** Abdul Rafey, Adnan Amin, Muhammad Kamran, Muhammad Imran Aziz, Varda Athar, Shah Iram Niaz, Luc Pieters

**Affiliations:** 1 Natural Products Research Lab (NPRL), Gomal Centre of Pharmaceutical Sciences, Faculty of Pharmacy, Gomal University, Dera Ismail Khan 29050, Pakistan; 2 Gomal Centre of Biochemistry and Biotechnology (GCBB), Gomal University, Dera Ismail Khan 29050, Pakistan; 3 Institute of Chemical Sciences (ICS), Gomal University, Dera Ismail Khan 29050, Pakistan; 4 Natural Products & Food Research and Analysis (NatuRA), Department of Pharmaceutical Sciences, University of Antwerp, Universiteitsplein 1, 2610 Antwerp, Belgium

**Keywords:** diabetes mellitus, advanced glycation end products (AGEs), protein cross link formation, *Punica granatum* peel, methylglyoxal

## Abstract

Diabetes mellitus (DM) is a global health concern that is associated with several micro- and macrovascular complications. We evaluated several important medicinal plant constituents, including polyphenols and flavonoids, for α-glucosidase inhibition, AGEs’ inhibitory activities using oxidative and no-oxidative assays, the inhibition of protein cross link formation, 15-lipoxydenase inhibition and molecular docking. The molecular docking studies showed high binding energies of flavonoids for transcriptional regulars 1IK3, 3TOP and 4F5S. In the α-glucosidase inhibition assay, a significant inhibition was noted for quercitrin (IC_50_ 7.6 µg/mL) and gallic acid (IC_50_ 8.2 µg/mL). In the AGEs inhibition assays, quercetin showed significant results in both non-oxidative and (IC_50_ 0.04 mg/mL) and oxidative assays (IC_50_ 0.051 mg/mL). Furthermore, quercitrin showed inhibitory activity in the non-oxidative (IC_50_ 0.05 mg/mL) and oxidative assays (IC_50_ 0.34 mg/mL). A significant inhibition of protein cross link formation was observed by SDS-PAGE analysis. Quercitrin (65%) and quercetin (62%) showed significant inhibition of 15-lipoxygenase. It was thus concluded that flavonoids and other polyphenols present in plant extracts can be effective in management of diabetes and allied co-morbidities.

## 1. Introduction

Diabetes mellitus (DM) is an ailment of the endocrine system that is associated with chronic insulin resistance and progressive exhaustion and death of β-cells in the pancreas that leads to hyperglycemia [1]. At present, 382 million people are currently diagnosed with diabetes globally with an expected massive increase to 600 million by the year 2035 [2]. About 4.9 million people die every year as a result of diabetes and 50% of this death toll is a consequence of diabetic complications [3]. In diabetes, several mechanisms initiate and impart injury to different vascular structures and repair mechanisms in such patients [4]. Thus, patients suffering from diabetes are susceptible to intense long-standing impediments like atherosclerosis, impaired wound healing, neuropathy, retinopathy, periodontitis, cataracts and nephropathy [5,6,7].

Advanced Glycation End products (AGE)s are the products produced via a chain of non-enzymatic reactions between reducing sugars, such as glucose, and the amino functionalities of proteins [8]. This reaction, also known as the Maillard reaction, starts when reducing sugars bind with the lysine side chain of a protein [9]. It primarily initiates the formation of a Schiff base that reshuffles into an intermediate product called an Amadori product. This is a crucial feature that permits the development of more complex and irreversible products called AGEs [10]. AGEs are yellow to brown, can show fluorescence, and can form unsolvable adducts with long-lived proteins, impairing their normal biological functions [11]. In this way, higher or uncontrolled glucose levels in diabetic patients contribute to the formation of AGEs and diabetic complications.

An association between inflammation, vascular complications and hyperglycemia has been observed in diabetic persons [12]. The pathogenicity of diabetes also involves the immune system. At the time a diabetes type 2 patient gets diagnosed, 50% of his pancreatic β-cells have already been destroyed [13]. Gradually, the pancreatic β-cells dysfunction increases and persistent hyperglycemia activates the immune system, thus leading to an increase in the inflammatory response. Thus, inflammation contributes to the pathogenesis of diabetes type 2 [14]. Moreover, in diabetes high blood plasma glucose and increased levels of free fatty acids could arouse inflammation processes that further increase glucose consumption through variations in the oxidative phosphorylation pathway [15]. It is also believed that oligomers of polypeptides (amyloid) of the pancreas might trigger inflammation through excitation of the NLRP3 inflammasome as well as production of IL-1β in type 2 diabetes [16].

Ayurvedic medicines are mainly based on natural products and may include plants, animals or minerals. They have centuries-old evidence of safety and efficacy [17]. Based on a survey of the Ayurvedic literature, some traditional medicinal plants including *Juglans regia*, *Salvadora persica*, *Syzygium aromaticum*, *Myristica fragrans*, *Punica granatum* and *Azadirachta indica* were selected for this investigation.

*Juglans regia* L. (Juglandaceae) is an important medicinal plant that is used for treatment of diabetes mellitus, inflammation and cancer [18,19,20]. It mainly contains ellagitannins, juglone and polyphenolic compounds. *Salvadora persica* L. (Salvadoraceae) is mainly linked with several biological activities including antimicrobial, antidiabetic, antirheumatic, anti-asthmatic, and anti-gonorrhea disorders [21]. This plant is a rich source of several classes of compounds including alkaloids, flavonoids, essential oils, sterols and terpenes [22,23]. *Syzygium aromaticum* (L.) Merr. & L.M.Perry (Myrtaceae) is a common Indian spice and is reported to contain several classes of compounds including polyphenols, triterpenes and an essential oil [24,25]. The plant is associated with several traditional medicinal claims including antioxidant, antimicrobial, antidiabetic and anticancer activities [26,27]. Likewise, *Myristica fragrans* Houtt. (Myristicaceae) is a traditional medicinal plant that has two main parts including mace and nutmeg [28]. Both parts of this plant are a rich source of polyphenols and essential oils containing myristicin, eugenol, and elemicin [29]. Traditional uses of nutmeg include antimicrobial, antidiabetic, carminative, hepatoprotective, anti-inflammatory and anti-rheumatic activities [30,31]. *Punica granatum* L. (Punicaceae) is an important medicinal plant that has many medicinal and nutritional benefits [32]. The peel part is a rich source of polyphenols, flavonoids and tannins including gallic acid, granatin A, granatin B, punicalin and punicalagin; quercetin, apigenin and anthocyanins such as pelargonidin [33]. Due to the presence of several important polyphenolic compounds, the peel is used for treatment of several diseases related to inflammation, diabetes, allergic reactions, and as an antioxidant [34,35]. Lastly, *Azadirachta indica* A. Juss (Meliaceae) is a commonly available plant in the sub-continent including India and Pakistan [36]. This plant is commonly used in Ayurvedic medicine for treatment of several ailments including bacterial and helminthic infections and diabetes mellitus [37]. Major chemical constituents include nimbidine, azadirachtin (azadirachtin A), salannol and salannin [38].

Keeping in mind the importance of these medicinal plants, we investigated the plant extracts and their major constituents for antidiabetic, antiglycation (oxidative and non-oxidative modes), and anti-inflammatory potentials using both computational and in vitro models. The study is important since it provides evidence-based information regarding designing a herbal formulation that can be helpful in the management of diabetes and associated health concerns including AGEs and inflammation. 

## 2. Results

### 2.1. Molecular Docking Results

**Figure 1:**
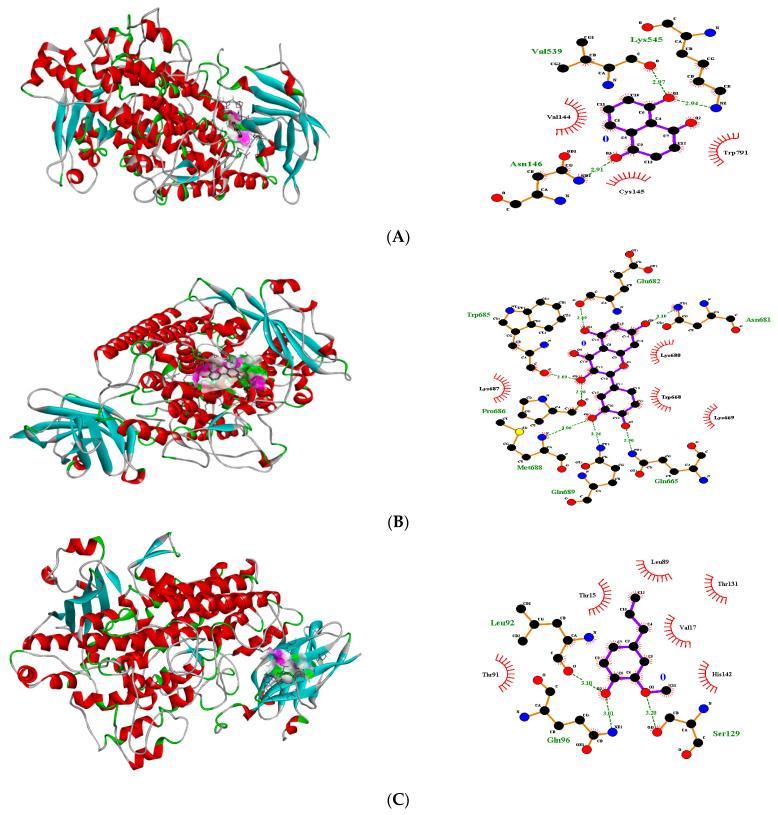
3D H-bonding interactions of juglone pose no. 3 (**A**), quercetin pose no, 4 (**B**); gallic acid pose no. 2 (**C**) with binding sites of transcriptional regulator 3TOP.

In the case of transcriptional regulator 3TOP (α-glucosidase), quercetin showed the best fit in the active pocket with pose no. 4 (Figure 1) (−7.9 ΔG (kJ mol^−1^). The number of H-bonding interactions was seven, including Pro686, Trp685, Glu682, Asn681, Gln665, Gln689, Met688 and the neighboring amino acid were Lys687, Lys680, Trp668, Lys669, involved in hydrophobic interactions (Table 1). Similarly, quercitrin presented a good fitting with pose no. 4 (−8.2 ΔG (kJ mol^−1^) with amino acids Ala163, Lys545, His548, Tyr544, Asp261, Asn146 (Table 1, Figure S3). Hydrophobic interactions were reported with Phe 264, Phe161, Cys145, Val539, Val544, Pro549, Asn788, and Val144 (Figure 1). Juglone and gallic acid also presented strong H-bonding interactions (Table 1, Figure 1), whereas caryophyllene-oxide α-humulene and *trans*-caryophyllene did not show any fitting within the active pocket of the transcriptional regulator (Table 1, Figures S1–S3). Docking images of all other compounds with transcriptional regulator 3TOP are shown in Supplementary Materials (Figures S1–S3).

**Figure 2:**
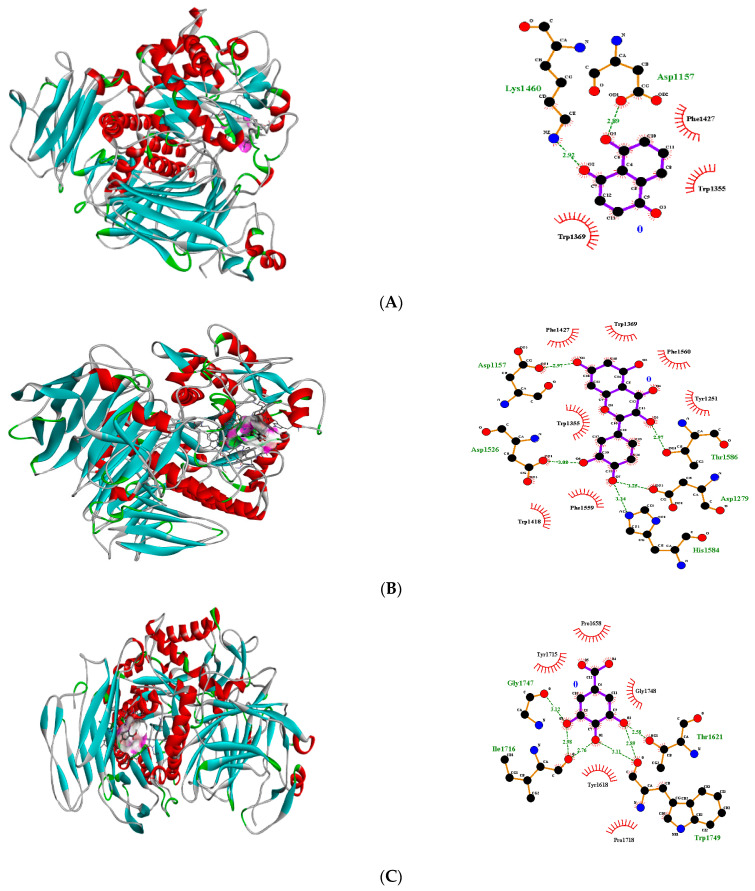
3D H-bonding Interactions of juglone pose no. 8 (**A**), quercetin pose no. 1 (**B**) and gallic acid pose no. 3 (**C**) with binding sites of transcriptional regulator 1IK3.

Molecular docking of all pure compounds on the active pockets of transcriptional regulator 1IK3 (15-lipoxygenase) was performed, and it was evident that flavonoids including quercitrin, quercetin and apigenin showed fitting within the active pocket with high binding free energy (ΔG (kJ mol^−1^) (Table 2). In the case of quercetin, the best fitting was noticed in pose 1 (−8.9 ΔG (kJ mol^−1^), where 5 H- bonding interactions were recorded with His1584, Asp1279, Thr1586, Asp1157 and Asp1526 (Table 2). The non-H bonding interactions/hydrophobic interactions were seen with neighboring amino acids Phe1559, Trp1418, Trp1355, Phe1427, Trp1369, Phe1560 and Tyr1251 (Figure 2). Juglone and gallic acid also presented strong H-bonding interactions (Table 2, Figure 2). Quercitrin showed 7 H bonding interactions (−8.2 ΔG (kJ mol^−1^) with Glu970, Asp969, Tyr967, Asp965, Trp985, Cys996 and Ser990 (Table 2). The other interactions including Vander Waal’s and *pi* sigma were also recorded with neighboring amino acids Gly992, Val993, Ser991, Phe995, Ala973 and Pro968 (Figure S6). 2-Phenyl-isothiocyanate, α-humulene and caryophyllene did not show any fitting within the active pocket of transcriptional regulator 3TOP. Docking images of all other compounds with transcriptional regulator 1IK3 are shown in Supplementary Materials (Figures S4–S6).

**Figure 3:**
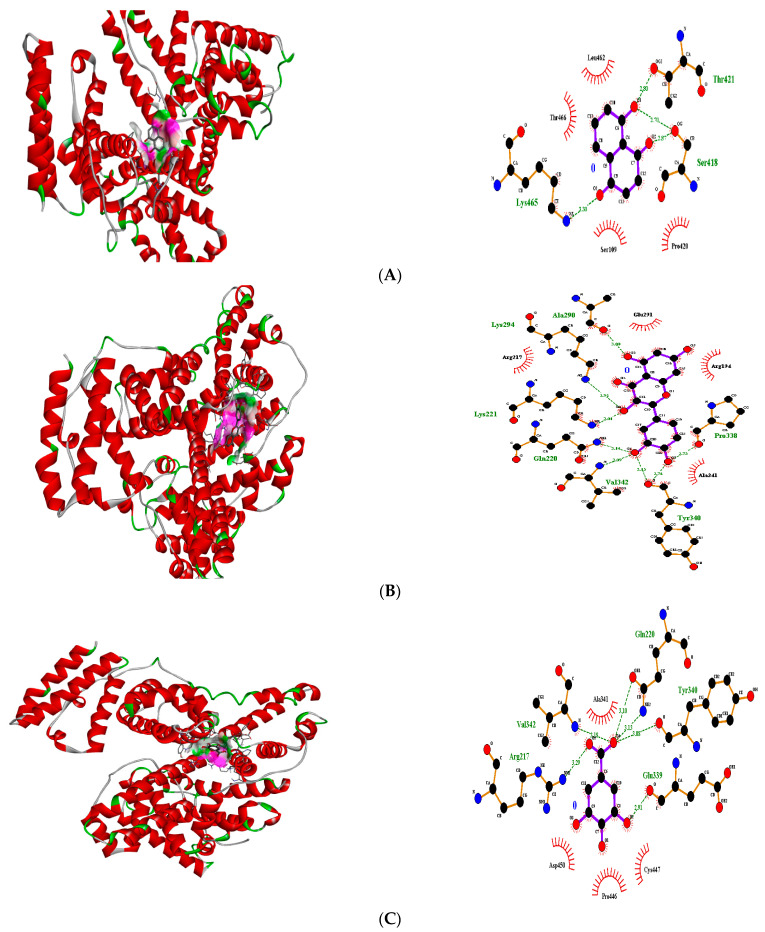
3D H-bonding interactions of juglone pose no. 5 (**A**), quercetin pose no. 4 (**B**) and gallic acid pose no. 1 (**C**) with binding sites of transcriptional regulator 4F5S (BSA).

Furthermore, the docking of ligands with the transcriptional regulator “4F5S” (BSA) yielded interesting results. Amongst flavonoids, quercetin showed the best fit in the active pocket of 4F5S with pose 4 (−7.3 ΔG (kJ mol^−1^) (Table 3). The number of contributing H-bonding interactions was seven, including Tyr340, Val342, Gln220, Lys221, Lys294, Ala290, Pro338, and the neighboring amino acids were Ala341, Arg217, and Glu291 that were involved in hydrophobic interactions (Figure 3). Juglone and gallic acid also presented strong H-bonding interactions (Table 3, Figure 3). Similarly, quercitrin presented good fitting by pose 5 (−7.2 ΔG (kJ mol^−1^) with amino acids Glu339, Lys294, Lys221, Ala290, Lys439, Pro338 (Table 3). The hydrophobic interactions were reported with Pro446, Arg217, Asp450, Tyr451 and Cys447 (Figure S9). Likewise, juglone showed the best fitting in the active pocket with pose 5 (−6.0 ΔG (kJ mol^−1^) with amino acids Thr421, Ser418 and Lys465 (Table 3). Terpenes including caryophyllene, caryophyllene oxide, α-humulene and 2-phenyl-isothiocyanate did not present any H-bonding affinity with BSA. Docking images of all other compounds with transcriptional regulator 4F5S are shown in Supplementary Materials (Figures S7–S9).

### 2.2. α-Glucosidase Assay

The extracts and pure compounds were assessed for their potential antidiabetic activities using the α-glucosidase assay. Amongst all tested extracts, only *Juglans regia* (61%) and the peel of *Punica granatum* (52%) showed the inhibition of α-glucosidase (Table 4). All other plants were considered inactive, since only mild inhibition was seen. In the case of 15-lipoxygenase activity, *Syzygium aromaticum* showed significant inhibition (70%), followed by *Myristica fragrans* (62%). A mild to moderate inhibition was seen for the other plant extracts.

With regard to the pure compounds, juglone (IC_50_ 7.6 µg/mL), quercitrin (IC_50_ 7.6 µg/mL) and gallic acid (IC_50_ 8.2 µg/mL) showed the inhibition of α-glucosidase. With regard to 15-lipoxygenase activity, quercitrin (65%), quercetin (62%) and α-humulene (60%) showed inhibition >50%. Inhibition results for other compounds are presented (Table 5).

### 2.3. Antiglycation Assays

In the AGEs inhibition assays, both oxidative (BSA-MGO) and non-oxidative (BSA-glucose) modes of inhibition were analyzed (Table 6). Among flavonoids, quercetin presented significant results in both the non-oxidative and (IC_50_ 0.04 mg/mL) oxidative assay (IC_50_ 0.051 mg/mL). Quercitrin showed a better inhibition in the non-oxidative (IC_50_ 0.09 mg/mL) than in the oxidative assay (IC_50_ 0.34 mg/mL) (Table 6). Apigenin (IC_50_ 0.45 mg/mL) was only active in the non-oxidative mode. Juglone has shown its activity in both the oxidative (IC_50_ 0.11 mg/mL) and non-oxidative (IC_50_ 0.06 mg/mL) assays, which reflects its potential use for diabetes and its complications (Table 6). All other tested compounds were recorded as inactive.

### 2.4. Protein Cross-Linking Assasy

**Figure 4:**
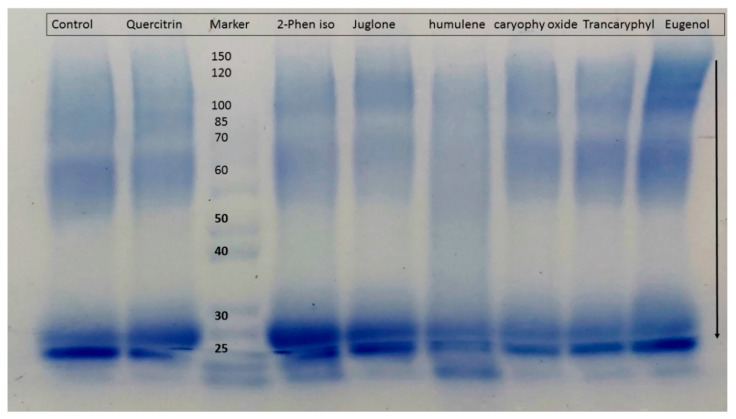
Gel showing BSA-MGO glycation with test compound (Control = BSA + MGO); Quercitrin = Quercitrin + MGO; Marker = Protein ladder; 2-Phen iso = 2-Phenylethylisothiocyanate + MGO; Juglone = Juglone + MGO; Humulene = α-Humulene + MGO; Caryophy Oxide = Caryophyllene oxide; Transcaryophyl = caryophyllene oxide + MGO; Eugenol = Eugenol + MGO.

The compounds were further tested for the inhibition of cross-link formation using both the non-oxidative (BSA-glucose) and oxidative (BSA-MGO) modes. SDS-PAGE was employed for measuring cross-linking. BSA incubation with either glucose or MGO produces small amounts of dimerization that is not visible with incubation of BSA alone. All tested compounds showed inhibition of cross-linked AGEs causing a decrease in intensity of dimerized (cross-linked) bands (Figure 4).

**Figure 5:**
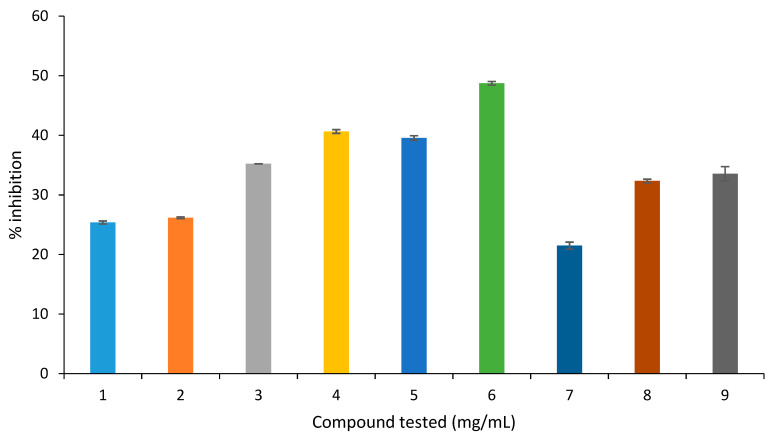
Inhibition of protein cross-link formation in the BSA-MGO assay (1 = eugenol, 2 = caryophyllene, 3 = caryophyllene oxide, 4 = α-humulene, 5 = juglone, 6 = 2-phenylethylisothiocyanate, 7 = quercetin, 8 = quercitrin, 9 = apigenin).

In the BSA-MGO assay, amongst all tested compounds, 2-phenylethylisothiocyanate presented the highest inhibition (48%) followed by α-humulene (40%), juglone (39%) and caryophyllene oxide (35%). The flavonoids showed a mild inhibition ranging from 21–34% (Figure 5). It was thus concluded that all tested compounds showed a mild inhibition of protein cross-link formation.

**Figure 6:**
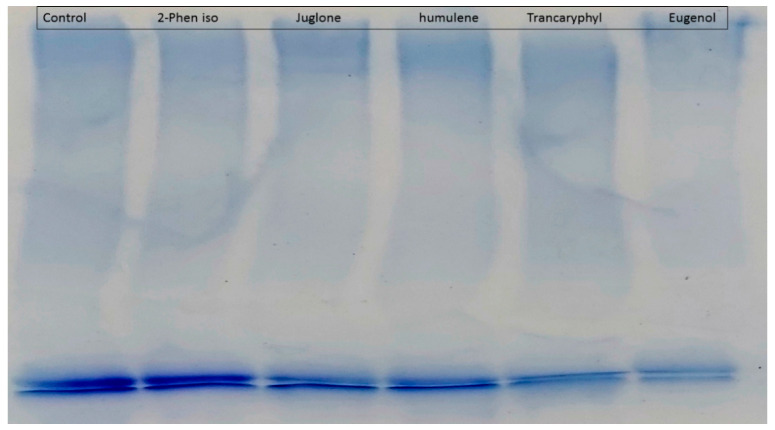
Gel showing BSA-Glucose glycation with tested compounds.

**Figure 7:**
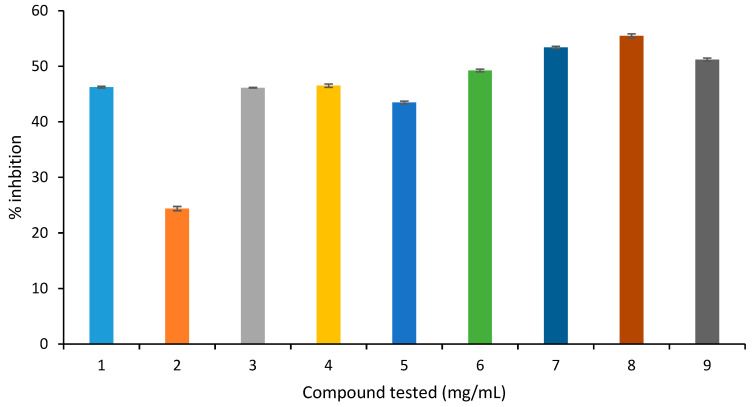
Inhibition of protein cross-link formation in the BSA-glucose assay (1 = eugenol, 2 = caryophyllene, 3 = caryophyllene oxide, 4 = α-humulene, 5 = juglone, 6 = 2-phenylethylisothiocyanate, 7 = quercetin, 8 = quercitrin, 9 = apigenin).

In the BSA-glucose assay (Figure 6), amongst all tested compounds, quercitrin presented the highest inhibition (55%), followed by quercetin (53%), and apigenin (51%). The flavonoids showed a mild inhibition ranging from 36–46% (Figure 7). It was thus concluded that all tested compounds showed a mild inhibition of protein cross-link formation.

## 3. Discussion

In this study, the effect of medicinal plant extracts and their major components on diabetes, glycation and inflammation was demonstrated using in vitro and in silico methods. Structural diversification of natural compounds enables multiple biological activities due to diverse mechanisms of action. Polyphenols and flavonoids possess several health benefits on account of their antioxidant, anti-inflammatory, and enzyme inhibiting properties [39]. Generally, the strong antioxidant potential of such compounds may contribute towards antidiabetic, antiglycation and anti-inflammatory activities [40,41]. 

α-Glucosidases are enzymes located at the intestinal lumen brush border that catalyze the hydrolysis of terminal, non-reducing α-1-4-linked glucose residues of disaccharides or oligosaccharides [42]. These enzymes are therefore helpful to facilitating carbohydrate absorption. Inhibitors of α-glycosidase can interrupt the digestion of carbohydrates to glucose and therefore they can be used for the treatment of type 2 diabetes [43]. In this study, flavonoids and polyphenols exhibited significant inhibition of α-glucosidase, which may be attributed to the presence of OH groups in the C-ring (flavonoids). It has been reported earlier that 3-hydroxylation of the C-ring facilitates the inhibitory activity against α-glucosidase [44]. The data is also in agreement with the molecular docking assessment, where strong H-bonding and hydrophobic interactions were observed for the transcriptional regulator gene for 3TOP (α-glucosidase).

Tissue inflammation is primarily related to immune system activation in diabetic patients [45]. Further phenotype conversion of macrophages from M2-type to M1-type is also very important in inflammatory conditions [46]. Lipoxygenase activation (12, 15-lipoxygenase) also plays a key role in the development of diabetes, and evidence has suggested that Lox inhibitors can greatly protect against diabetes [47]. Our results indicated that flavonoids and polyphenolic compounds possess significant inhibition of 15-lipopxygenase. This is further supported by molecular docking results, which indicate strong H-bonding, and hydrophobic interactions with transcription regulator 3TOP. This could be due to *pi*-interaction of the bond linking the B and C rings [40] that gives a near planar region of these two rings. Such structures (like flavonoids) easily enter the hydrophobic pockets in enzymes and can subsequently increase their inhibitory effect. Thus the C2–C3 double bond of flavonoids is crucial for their anti-inflammatory activity [48].

AGEs build-up in the body can activate several signaling pathways through receptors and thus interrupt various biological activities and cellular functions that finally lead to cell death [49]. Various mechanisms have been documented to explain AGEs synthesis in the human body including oxidative and non-oxidative modes [50]. In this investigation, flavonoids are reported with antiglycation activities in both oxidative and non-oxidative models that may be due to their radical scavenging properties, thus a delay in the progression of glycation is expected [51,52]. The cross-linking AGEs possess strong affinity towards diverse proteins and therefore are resistant to degradation. This leads to further toxicity to the human body [53]. Furthermore, flavonoids also showed inhibition of protein cross-link formation. It was evident from earlier findings that flavonoids and polyphenols inhibit cross-link formation because of antioxidant properties and some other mechanisms [54]. Thus, radical scavenging and inhibition of cross-link formation may provide a protective effect against hyperglycemia-mediated damaging effects on proteins [55]. Our in silico results also supported this fact by indicating strong H-bonding interactions of flavonoids and polyphenols with transcriptional regulator 4F5S (BSA).

## 4. Materials and Methods

### 4.1. Plant Materials

A detailed literature review of Ayurvedic medicinal plants and their formulations was performed and medicinal plants were purchased from local herbal markets and identified by a taxonomist in the Institute of Biological Sciences, Gomal University, Pakistan. The plants used during this investigation are shown in the Supplementary Materials (Table S1). The plants were analyzed as reported before using HPLC-DAD, GC-MS, LC-QTOF-MS [56], and major components are highlighted in the Supplementary Materials (Figure S1).

### 4.2. Extraction and Drying

Plant material was dried in an oven below 40 °C. Next, the plant material was thoroughly grinded followed by cold maceration in 90% methanol. Solvent evaporation was accomplished by a rotary evaporator (Büchi, Flawil, Switzerland), and stored at 4 °C until use. The essential oils were obtained using a Clevenger apparatus (hydrodistillation) and fixed oils were obtained using cold pressing [56].

### 4.3. Chemicals Reagents and Solvents

15-LOX (lipoxygenase), α-glucosidase, DPPH (2,2-diphenyl-1-picrylhydrazyl radical), bovine serum albumin (BSA), trichloroacetic acid (TCA), methylglyoxal (MGO) and D-glucose were purchased from Merck, (Dorset, UK), and Oxoid (Hampshire, UK), whereas NaN_3_ was purchased from DaeJung (Siheung-si, Korea). Chemical for Gel analysis including Coomassie blue, Tris–HCl, sodium dodecyl sulphate, 2-mercaptoethanol, Glycerine, bromophenol blue were purchased from Sigma Aldrich, St. Louis, MO, USA. The standard compounds included aminoguanidine (≥98.5%, Sigma Aldrich, St. Louis, MO USA), eugenol (≥99%, Fluka, Riedstr, Germany), 2-phenylethylisothiocyanate (>99%, Sigma Aldrich, St. Louis, MO, USA), juglone (≥98.5%, Santa Cruz Biotechnology, Santa Cruz, CA, USA), quercetin (≥99%, Sigma Aldrich, St. Louis, MO, USA), quercitrin (≥85%, Sigma Aldrich, St. Louis, MO, USA), *trans*-caryophyllene (≥98.5%, Fluka, , Riedstr, Germany), α-humulene (>98%, Extrasynthese, Genay, France), caryophyllene-oxide (≥99%, Fluka Honeywell, Seelze, Germany) and apigenin (≥95.5% Sigma Aldrich, St. Louis, MO, USA) (structures are shown in the Supplementary Materials).

### 4.4. Molecular Docking

For molecular docking studies, the X-ray crystallographic structures of the transcriptional regulators 3TOP [57] and 1IK3 [58] were taken from the Protein Data Bank (PDB) and active pocket dimensions for each protein were checked using the CAST*p* 3.0 online tool. The optimization of transcriptional regulators was performed using DS Visualizer 2.0 [59]. Furthermore, the structures of all the phytoconstituents were downloaded from the Pubchem database and PDB files were generated in DS Visualizer 2.0 [59]. The molecular docking was performed using Lamarckian Genetic Algorithm embedded in AutoDock v 4.2 [60]. A total number of nine poses were generated (for each target) and grouped according to their RMSD values. Every set was prudently checked in Discovery Studio Visualizer and presumed binding modes were highlighted for further analysis. Best docked structures based on the binding energy scores (ΔG) and H- binding were chosen for further analysis. The hydrogen bonding and hydrophobic interactions between ligand and protein were calculated by Ligplot^+^ and DS Visualizer 2.0.

### 4.5. Antidiabetic Assays

#### 4.5.1. α-Glucosidase Inhibition Assay

The α-glucosidase inhibition experiment was accomplished by using a modified method [61]. Initially, the enzyme solution (from *Saccharomyces cerevisiae*) (0.2 units/mL dissolved in 0.1 M phosphate buffer; pH 6.8) was mixed with the test sample (1 to 0.039 mg/mL) and incubated in an oven (37 °C for 10 min). After incubation, the substrate (*p*-nitrophenyl-α-D-glucopyranoside; 0.29 mM) was added to the enzyme and test sample solution and incubated for another 30 min (37 °C). The reaction was halted by adding Na_2_CO_3_ (100 µL, 200 mM stock) to this mixture and absorbance was noted at 400 nm. Acarbose was used as positive control.

The percentage of inhibition was determined using the following formula:

% Inhibition = [1 − absorbance of test sample/absorbance of control] × 100

#### 4.5.2. AGEs Assay (BSA-Glucose Assay)

The AGEs assay was accomplished by using a standard protocol [62,63]. The protein source (bovine serum albumin, BSA) (10 mg/mL; 135 µL) was mixed with D-glucose solution (500 mM, 135 µL in phosphate buffer; 50 mM, pH 7.4), NaN_3_ (sodium azide; 0.02%) and test samples (various concentrations). The reaction mixtures were kept at 60 °C for 1 week to facilitate glycation. Finally, trichloroacetic acid (10 µL, 100%) was added to it to stop the reaction and precipitate the unbound material. The supernatant was removed and the pellet was dissolved in alkaline phosphate buffer saline (137 mM NaCl, 8.1 mM Na2HPO4, 2.68 mM KCl, 1.47 mM KH2PO4, pH10). Finally the fluorescence intensity (ex 370/emis 440; ex 335/emis 385 nm) was recorded on a spectrofluorometer (FLx800, BioTek, Winooski, USA). Aminoguanidine was used as positive control. The control samples used for the experiment were prepared using the same protocol without the test sample.

The AGEs inhibition was calculated as

% inhibition = {1 − [Fluo(BSA + glucose + test substance) − Fluo (BSA + test substance)]/[Fluo (BSA + glucose) − Fluo(BSA)]} × 100

where Fluo is the fluorescence intensity. The IC_50_ was calculated using MS Excel.

#### 4.5.3. BSA-MGO Assay

In this oxidative glycation assay, the protein source (BSA) was mixed (10 mg/mL, 135 µL) with methylglyoxal (5.75 mM, 135 µL) and liquefied in phosphate buffer (50 mM, pH 7.4) and NaN_3_ (sodium azide; 0.02%). The test samples were added to this reaction mixture and stored at 37 °C for a week. The change in fluorescence intensity (ex 370/emis 440; ex 335/emis 385 nm) was recorded on a spectrofluorometer (FLUOstar Omega^®^, BMG Lab Tech, Aylesbury, UK). Aminoguanidine and quercetin were used as positive controls. The control samples used for the experiment were prepared using the same protocol without a test sample.

The AGEs inhibition was determined as

% inhibition = {1 − [(Fluo (BSA + MGO + test substance) − Fluo (BSA + test substance)]/[(Fluo (BSA + MGO) − Fluo (BSA)]} × 100

where Fluo is the fluorescence intensity. The IC_50_ was calculated using MS Excel.

#### 4.5.4. Protein Cross-Linking Assay

Protein cross-linked by AGEs were observed by SDS-PAGE (sodium dodecyl sulphate-polyacrylamide gel electrophoresis) [64]. In this experiment, gels (10%) were made and stained with dye (0.25% Coomassie blue). The protein samples (6 µL) were diluted with Tris–HCl buffer (3.75 mL, 0.05 M; pH 6.8) also comprising sodium dodecyl sulphate (150 µL, 10% *w*/*v*), 2-mercaptoethanol 1% (*v*/*v*) and glycerin 20% *v*/*v*), followed by boiling for 5 min. The ladder (12 μL) was loaded into the well on gel preceded by the loading of bromophenol blue (2 μL). After loading, the electrophoresis was accomplished by using the Mini-Protean^®^ Tetra Cell apparatus (Bio-Rad, UK). Afterwards, the gels were stained in a solution containing Coomassie blue [0.25% (*w*/*v*)], methanol [50% (*v*/*v*)] and acetic acid [10% (*v*/*v*)]. 

#### 4.5.5. SDS-PAGE Gels Image Analysis

The developed gel was stained with Coomassie blue and images were obtained using GelDoc. Finally, the Image J tool was used for the determination of integrated density (IntDen). The integrated density was employed further for the determination of percentage inhibition. The integrated density (ID) was determined as follows:

Integrated density (ID) = N × (mean − background)

where N is the number of pixels in the selection and the background is the modal grey value (most common pixel value) after smoothing the histogram.

The percentage inhibition of cross-linked AGEs was determined using the formula:

% inhibition = 100 × (ID without inhibitor − ID with inhibitor)/ID without inhibitor

### 4.6. 15-Lipoxygenase Assay

The anti-inflammatory activity of test samples was evaluated using a standard protocol [65]. To the enzyme solution (200 units/mL; 487.5 µL), different concentrations of test sample (12.5 µL (2–0.062 mM) in DMSO were added and incubated for 5 min at 37 °C. The absorbance was recorded immediately after the addition of substrate (500 µL of substrate (250 mM linoleic acid in 0.2 M borate buffer, pH 9) and after every min up to 5 min at 234 nm by using a UV spectrophotometer (UV-1601, SHEMADZU, Kyoto, Japan).

The percentage inhibition of enzyme activity was calculated as follows:

% inhibition = ([ΔA1/Δt] − [ΔA2/Δt]/(ΔA1/Δt) × 100

where ΔA1/Δt and ΔA2/Δt are the increase rate in absorbance at 234 nm for a sample without test substance and with test substance, respectively.

### 4.7. Statistical Analysis

All experiments were performed in triplicate and results were expressed as mean ± SD. 

## 5. Conclusions

Diabetes, a very prevalent metabolic disorder, is an important health problem worldwide. Persistent hyperglycemia results in the development of inflammation and several life threatening complications due to the production of AGEs. Various strategies are being used by researchers to introduce new treatment options that have a dual effect i.e., both blood glucose lowering and anti-AGEs potential. Traditional medicinal plants are considered as effective and reliable alternatives of conventional medical therapy due to proven safety and efficacy. In this investigation, selected medical plants and their major constituents were analyzed using in silico and in vitro models. Interactions of flavonoids and polyphenols were observed with transcription regulators 1IK3, 3TOP and 4F5S. Further in vitro assays presented anti diabetic, antiglycation, and anti-inflammatory activities of constituents including juglone, quercetin, quercitrin, apigenin and 2-phenylethylisothiocyanate from *Juglans regia*, *Punica granatum* and *Myristica fragrans.* Thus, it was concluded that these plant species may be considered as candidates for the management of diabetes mellitus and co-morbidities occurring due to AGEs.

## Figures and Tables

**Table 1:** Docking score, H and non-H bonding interactions of test compounds for transcriptional regulator 3TOP (α-glucosidase).

	Compound	Binding Free Energy ΔG (kJ mol^−1^)	Pose No	H Bond	H Bond Interaction Residues	Neighbor Interacting Residues
	**3TOP**					
**1**	Eugenol	−5.3	3	3	Ser129, Gln96, Leu92	Thr91, Thr15, Leu89, Thr131, Val17, His142
**2**	Caryophyllene	−7.6	1	0	0	Lys545, Val539, Cys145, Asn146, Val144, His548, Trp791, Pro789, Asn788, Pro549, Tyr544
**3**	Caryophyllene oxide	−6.6	1	0	-	Leu154, Glu 199, Trp198, Arg200, Asn186, Ser157, Phe155,
**4**	α-Humulene	−6.4	1	0	-	Lys388, Val589, Asp592, Tyr512, Pro432, Val588, Asp431, Lys388
**5**	Juglone	−6.3	3	3	Asn146, Val539, Lys545	Cys145, Trp791, Val144
**6**	2-Phenylethyl-isothiocyanate	−5.5	1	1	Lys545	Pro549, Trp791, Asp 787, Val144, Val539
**7**	Quercetin	−7.9	4	7	Pro686, Trp685, Glu682, Asn681, Gln665, Gln689, Met688.	Lys687, Lys680, Trp668, Lys669
**8**	Quercitrin	−8.2	4	6	Ala163, Lys545, His548, Tyr544, Asp261, Asn146	Phe 264, Phe161, Cys145, Val539, Val544, Pro549 Asn788, Val144,
**9**	Apigenin	−7.4	5	4	Glu197, Asn556, Thr274, Lys 278	Tyr257, Arg260, Val256, Leu258, Tyr275, Ala263

**Table 2:** Docking score, H- and non-H bonding interactions of test compounds for transcriptional regulator 1IK3 (15-lipoxygenase).

	Compound	Binding Free Energy ΔG (kJ mol^−1^)	Pose No	H Bond	H Bond Interaction Residues	Neighbor Interacting Residues
	**1IK3**					
**1**	Eugenol	−6.1	2	2	Glu1400, Glu1397	Phe1289, Pro1405, Asn1404, Leu1412, Leu1401,
**2**	Caryophyllene	−6.3	1	0	0	Asp1157, Trp1355 Asp1526, Phe1560, Tyr1251, Thr1586 Trp1369, Phe1427,
**3**	Caryophyllene oxide	−6.1	1	2	Gln1372, Arg1377	Ile1587, Tyr1251, Ile1280, Asp1281, Gln 1286, Asp1357
**4**	α-Humulene	−6.1	1	0	0	Pro1159, Phe1427, Asp1157, Asp1526 Phe1560, Trp1355 Trp1369
**5**	Juglone	−5.2	8	2	Asp1157, Lys1460	Phe1427, Trp1355, Trp1369
**6**	2-Phenylethyl-isothiocyanate	−4.4	1	0	0	Pro1327, Pro1329, Glu1397, Glu1400, Leu1401, Phe1289,
**7**	Quercetin	−8.9	1	5	His1584, Asp1279, Thr1586, Asp1157 Asp1526	Phe1559, Trp1418, Trp1355, Phe1427 Trp1369, Phe1560, Tyr1251,
**8**	Quercitrin	−8.2	4	7	Glu970, Asp969, Tyr967, Asp965 Trp985, Cys996 Ser990	Gly992, Val993, Ser991 Phe995, Ala973, Pro968
**9**	Apigenin	−7.6	7	5	Glu1284, Glu1397, Arg1333, Glu1400, Leu1291	Phe1289, Pro1239, Thr1290,

**Table 3:** Docking score, H- and non-H bonding interactions of test compounds with transcriptional regulator 4F5S (BSA).

	Compound	Binding Free Energy ΔG (kJ mol^−1^)	Pose No	H Bond	H Bond Interaction Residues	Neighbor Interacting Residues
	**4F5S**					
**1**	Eugenol	−5.3	1	1	Glu125	Phe133, Tyr137, Tyr160, Leu115, Leu122,
**2**	Caryophyllene	−7.6	1	0	0	Val481, Leu346, Ala212, Ala209, Arg208, Phe205, Leu480,
**3**	Caryophyllene oxide	−5.7	1	0	0	Gln203, Ile202, Cys245, His246, Lys242,
**4**	α-Humulene	−6.9	1	0	0	Leu112, Glu125, Lys136, Phe133, Tyr160, Tyr137, Glu140, Leu115, Leu122
**5**	Juglone	−6.0	5	3	Thr421, Ser418, Lys465	Pro420, Ser109, Thr466, Leu462,
**6**	2-Phenylethyl-isothiocyanate	−4.7	1	0	0	Lys132, Lys131, Leu24, Gly21, Val43, Lys20
**7**	Quercetin	−7.3	4	7	Tyr340, Val342, Gln220, Lys221, Lys294, Ala290, Pro338,	Ala341, Arg217, Glu291,
**8**	Quercitrin	−7.2	5	6	Glu339, Lys294, Lys221, Ala290, Lys439, Pro338,	Pro446, Arg217, Asp450, Tyr451, Cys447,
**9**	Apigenin	−7.8	3	4	Lys431, Arg458, Asn457, Leu454,	Arg435, Leu189, His145, Arg196, Ala193,

**Table 4:** α-Glucosidase and 15-Lipoxygenase assay of selected medicinal plants.

		Assay Type	
	Name	α-Glucosidase *	15-Lox Assay *
**1**	*Juglans regia*	61.0 ± 0.1%	14.0 ± 0.2%
**2**	*Syzygium aromaticum*	Inactive	70.0 ± 0.1%
**3**	*Eruca sativa*	Inactive	45.0 ± 0.1%
**4**	*Myristica fragrans*	Inactive	62.00 ± 0.04%
**5**	*Punica granatum*	52.0 ± 0.1%	10.00 ± 0.04%
**6**	*Azadirachta indica*	Inactive	45.0 ± 0.1%
	Standard	72.0 ^a^ ± 0.1%	62.0 ^b^ ± 0.1%

* 60 µg/mL; ^a^ Acarbose (0.01 mM), ^b^ Rutin (12.5 µg/mL).

**Table 5:** α-Glucosidase and 15-LOX inhibitory activity of test compounds.

	Compound	Assay Type	
		α-Glucosidase (IC_50_) (µg/mL)	15-LOX Assay (% Inhibition)
**1**	Eugenol	Inactive	51.0 ± 0.2% ^4^
**2**	Caryophyllene	Inactive	Inactive ^6^
**3**	Caryophyllene oxide	Inactive	50.0± 0.1% ^2^
**4**	α-Humulene	Inactive	60.0 ± 0.1% ^3^
**5**	Juglone	5.7 ± 0.1	57.0 ± 0.1% ^1^
**6**	2-Phenylethylisothiocyanate	28.9 ± 0.1	Inactive ^5^
**7**	Quercetin	21.2 ± 0.1	62.0 ± 0.1% ^7^
**8**	Quercitrin	7.6 ± 0.1	65.0± 0.1% ^9^
**9**	Apigenin	24.4 ± 0.1	53.0 ± 0.1% ^8^
**10**	Standard	6.49 ± ^a^ 0.02	62.0 ± 0.1% ^b^

^1^ 2.5 µg/mL, ^2^ 12.5 µg/mL, ^3^ 13.0 µg/mL, ^4^ 83.0 µg/mL, ^5^ 25 µg/mL, ^6^ 25 µg/mL, ^7^ 25 µg/mL, ^8^ 14.1 µg/mL, ^9^ 16.2 µg/mL. ^a^ Acarbose (µg/mL), ^b^ Rutin (12.5 µg/mL).

**Table 6:** Antiglycation assay of compounds.

	Compound	Protein Glycation	
		BSA-Glucose (IC_50_) (µg/mL)	BSA-MGO (IC_50_) (µg/mL)
**1**	Eugenol	0.040 ± 0.007	Inactive
**2**	Caryophyllene	Inactive	Inactive
**3**	Caryophyllene oxide	Inactive	Inactive
**4**	α-Humulene	Inactive	Inactive
**5**	Juglone	0.060 ± 0.004	0.11 ± 0.04
**6**	2-Phenylethylisothiocyanate	Inactive	Inactive
**7**	Quercetin	0.040 ± 0.004	0.050 ± 0.005
**8**	Quercitrin	0.090 ± 0.008	0.34 ± 0.06
**9**	Apigenin	0.45 ± 0.02	Inactive
**10**	Standard ^1^	0.030 ± 0.001	1.02 ± 0.21

^1^ Rutin.

## Data Availability

The data presented in this study are available in this article and also as Supplementary Materials.

## Supplementary Materials

The following Tables and Figures are available online at https://www.mdpi.com/article/10.3390/molecules27196715/s1, Table S1. Ayurvedic medical plants and their major constituents. Figure S1. Structure of test compounds. Figure S2. 3D H-bonding interactions of 2-phenyl isothiocyanate pose no. 1 [1], Apigenin pose no. 5 [2] caryophyllene oxide pose no. 1 [3] with binding sites of transcriptional regulator 1IK3. Figure S3. 3D H-bonding interactions of eugenol pose no. 3 [4], α-humulene pose no. 1 [5] with binding sites of transcriptional regulator 1IK3. Figure S4. 3D H-bonding interactions of quercitrin pose no. 4 [6] and caryophyllene pose no. 1 [7] with binding sites of transcriptional regulator 1IK3. Figure S5. 3D H-bonding Interactions of 2-phenylethylisothiocyanate pose no. 1 [8], apigenin pose no. 1 [9]; caryophyllene oxide pose no. 1 [10] with binding sites of transcriptional regulator 3TOP. Figure S6. 3D H-bonding interactions of eugenol pose no. 2 [11] and α-humulene pose no. 1 [12] with binding sites of transcriptional regulator 3TOP. Figure S6. 3D H-bonding interactions of quercitrin pose no. 4 [13] and caryophyllene pose no. 1 [14] with binding sites of transcriptional regulator 3TOP. Figure S7. 3D H-bonding interactions of 2-phenylethylisothiocyanate pose no. 1 [15], apigenin pose no. 3 [16] and caryophyllene oxide pose no. 1 [17] with binding sites of transcriptional regulator 4F5S. Figure S9. 3D H-bonding interactions of quercitrin pose no. 5 [20] and caryophyllene pose no. 1 [21] with binding sites of transcriptional regulator 4F5S [66,67,68,69].

## Sample Availability

Samples of the compounds are not available from the authors.
