# Supplementary material for: Evaluation of Major Constituents of Medicinally Important Plants for Anti-Inflammatory, Antidiabetic and AGEs Inhibiting Properties: In Vitro and Simulatory Evidence
Source: Molecules. 2022 Oct 9;27(19):6715. doi: 10.3390/molecules27196715 (PMC9571302; doi:10.3390/molecules27196715)
Supplement: Supplementary file 1 [file molecules-27-06715-s001.zip › molecules-1885592-supplementary.pdf]

Supplementary data

Table S1. Ayurvedic medical plants and their major constituents

**Plant species**	**Major components**	**References**
Juglans regia L.	Juglone	(Raafa, 2018)
Syzygium aromaticum (L.) Merr. & L.M.Perry	Eugenol, caryophyllene, α-humulene, caryophyllene oxide	(Alma *et al.*,2007)
Eruca sativa Mill.	2-Phenylethylisothiocyanate	(Khoobchandani *et al.*, 2010)
Myristica fragrans Houtt	Caryophyllene oxide, palmitic acid , 2-Phenylethylisothiocyanate	(Shafiq et al., 2016)
Punica granatum L.	Gallic acid, apigenin, quercetin, pelargonidin, cyanidin, punicalin, punicalagin, granatin A, granatin B.	(Singh *et al.*, 2018)
Azadirachta indica A. Juss	Nimbidine, azadirachtin (azadirachtin A), salannol, salannin	(Biswas *et al.*, 2002)

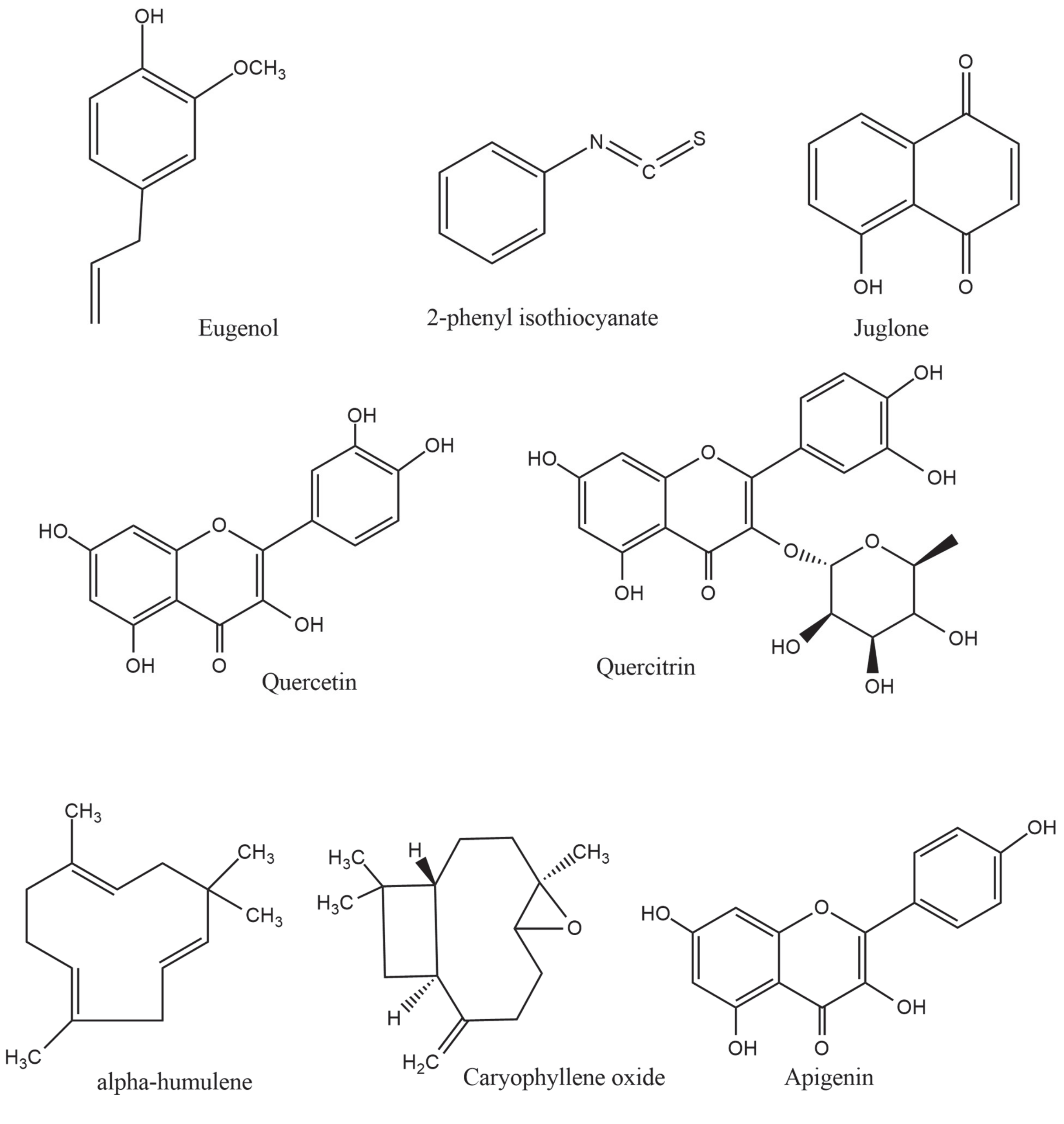

Figure S1. Structure of tested compounds including Eugenol($C_{10}H_{11}O_2$), 2-Phenyl isothiocyanate (C_7H_5NS), Juglone ($C_{10}H_6O_3$), Quercetin($C_{15}H_{10}O7$), Quercitrin ($C_{21}H_{20}O_{11}$), Alpha humulene ($C_{15}H_{22}$), Caryophyllene oxide ($C_{15}H_{24}O$), Apigenin ($C_{15}H_{10}O_6$).

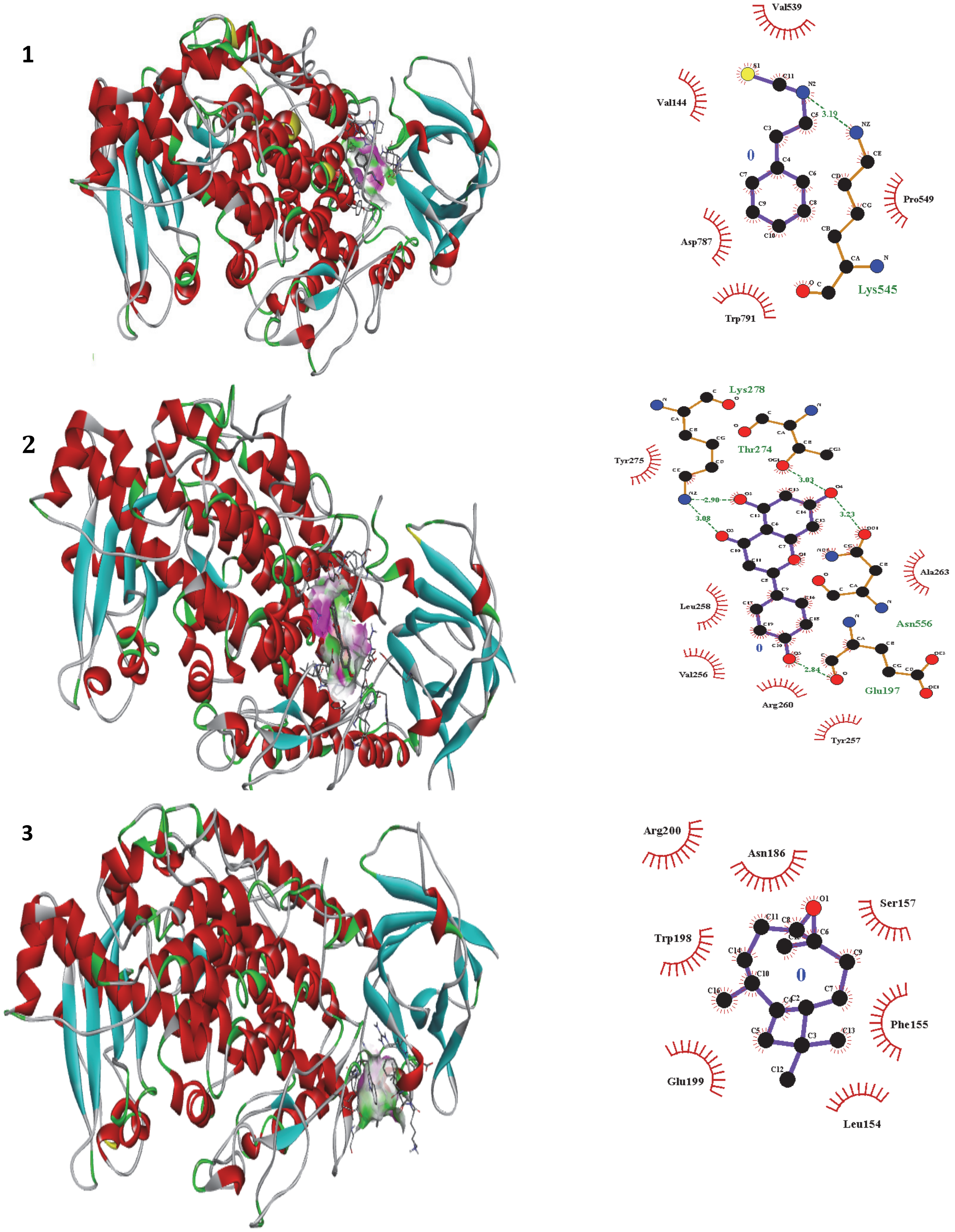

Figure S2. 3D H-bonding interactions of 2-phenyl isothiocyanate pose no. 1 [1], apigenin pose no. 5[2] caryophyllene oxide pose no. 1 [3] with binding sites of transcriptional regulator **1IK3.**

4

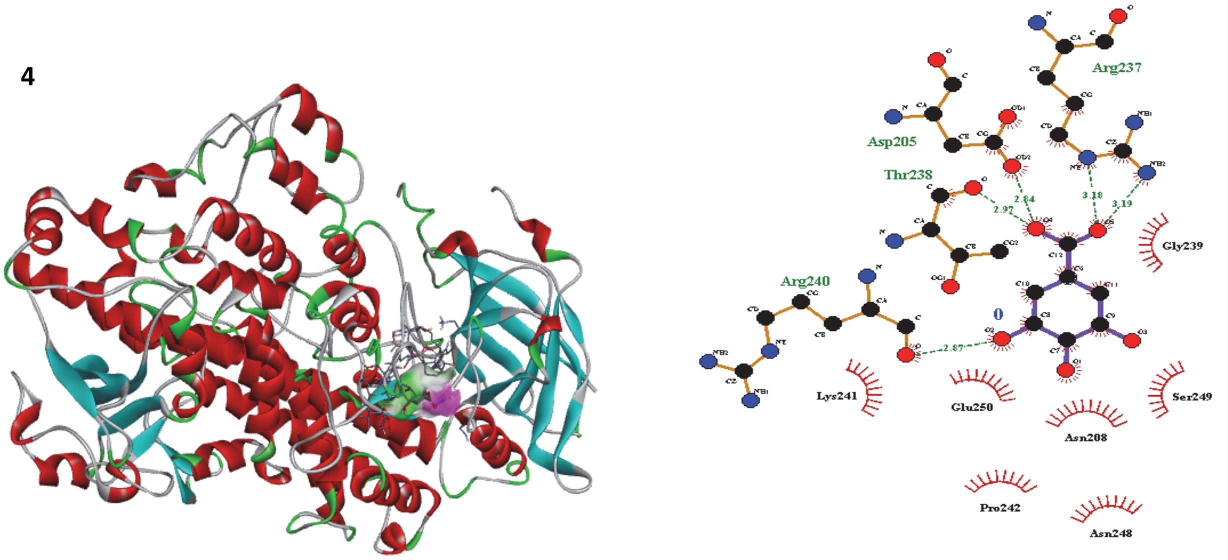

5

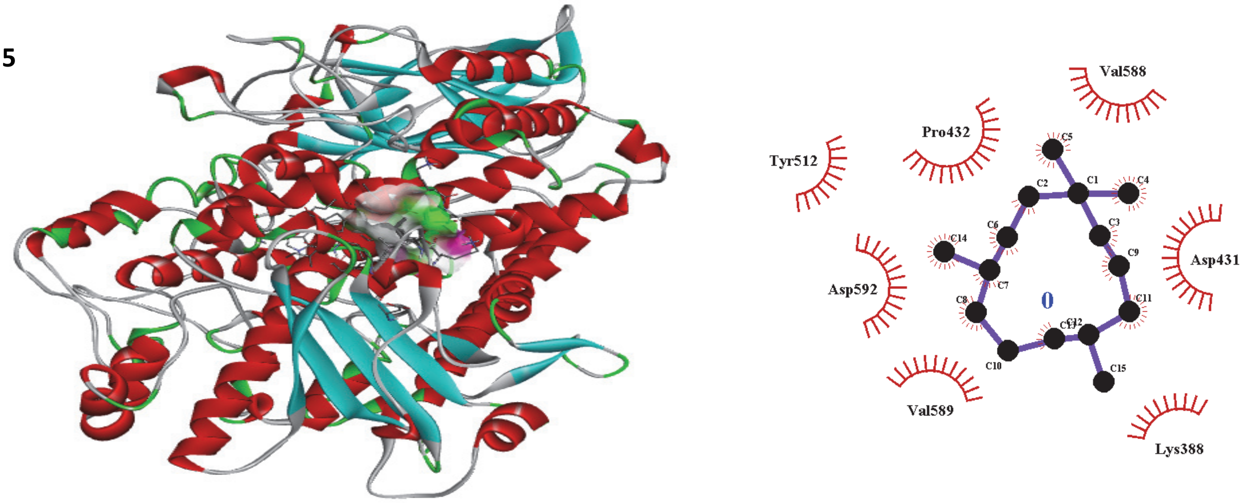

Figure S3. 3D H-bonding interactions of Eugenol pose rank 3 [4], α-humulene pose no. 1 [5] with binding sites of transcriptional regulator **1IK3**

6

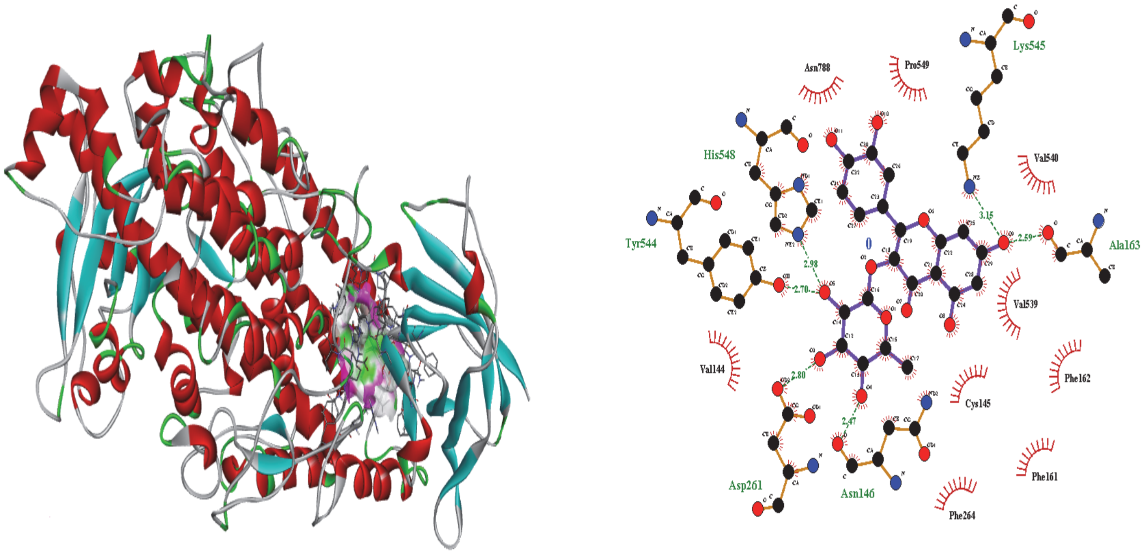

7

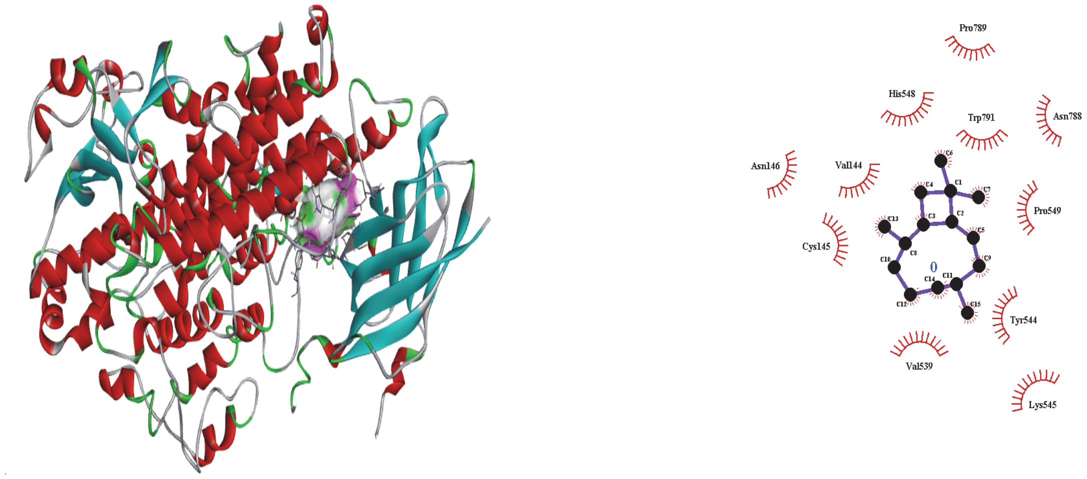

Figure S4. 3D H-bonding interactions of Quercitrin pose no. 4 [6] and transcaryphyllene pose no.1 [7] with binding sites of transcriptional regulator **1IK3.**

8

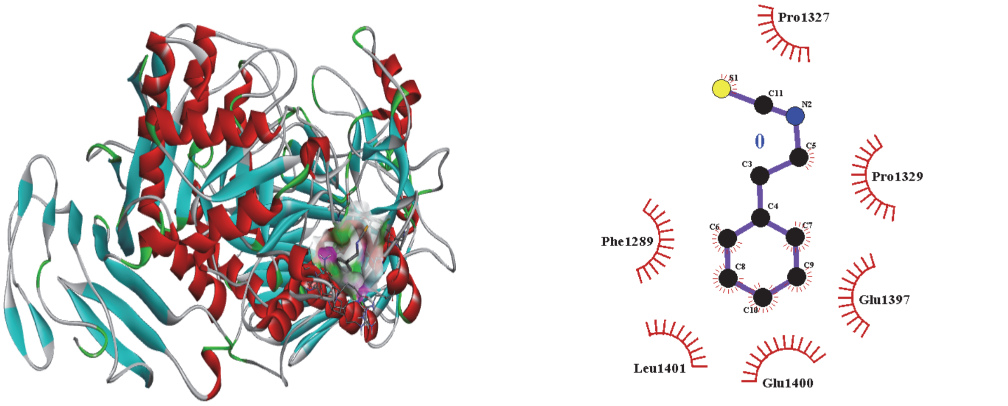
Pro1327
S1
C11
N2
0
C5
C3
Pro1329
C4
Phe1289
C6
C7
C8
C9
C10
Glu1397
Leu1401
Glu1400

9

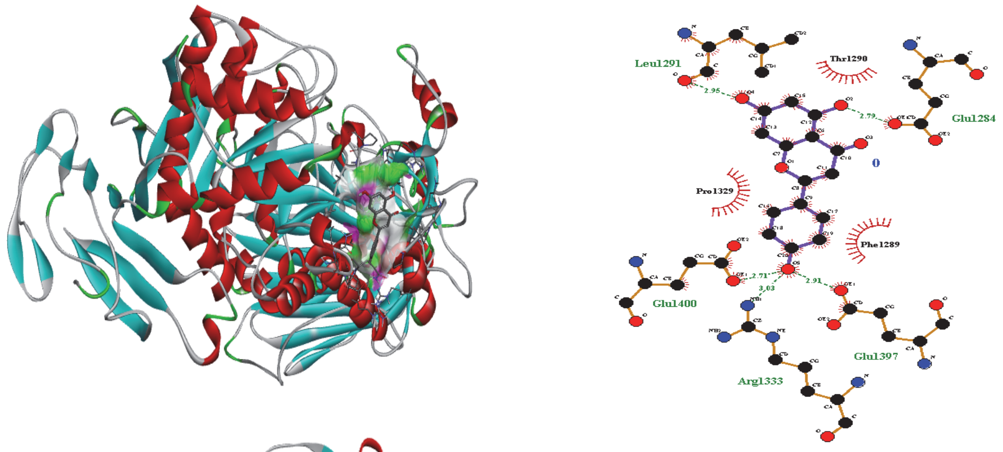
Leu1291
Thr1290
2.95
2.79
Glu1284
0
Pro1329
Phe1289
2.71
3.03
2.91
Glu1400
Arg1333
Glu1397

10

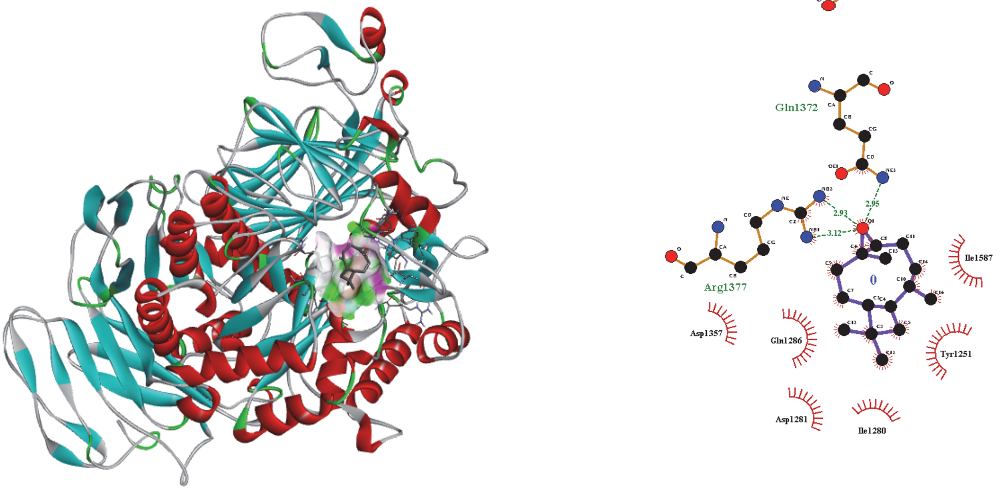
Gln1372
2.95
2.93
3.12
Ile1587
0
Arg1377
Asp1357
Gln1286
Tyr1251
Asp1281
Ile1280

Figure S5. 3D H-bonding interactions of 2-phenylisothiocyanate pose no. 1 [8], apigenin pose no. 1 [9]; caryophyllene oxide pose no. 1 [10] with binding sites of transcriptional regulator **3TOP.**

11

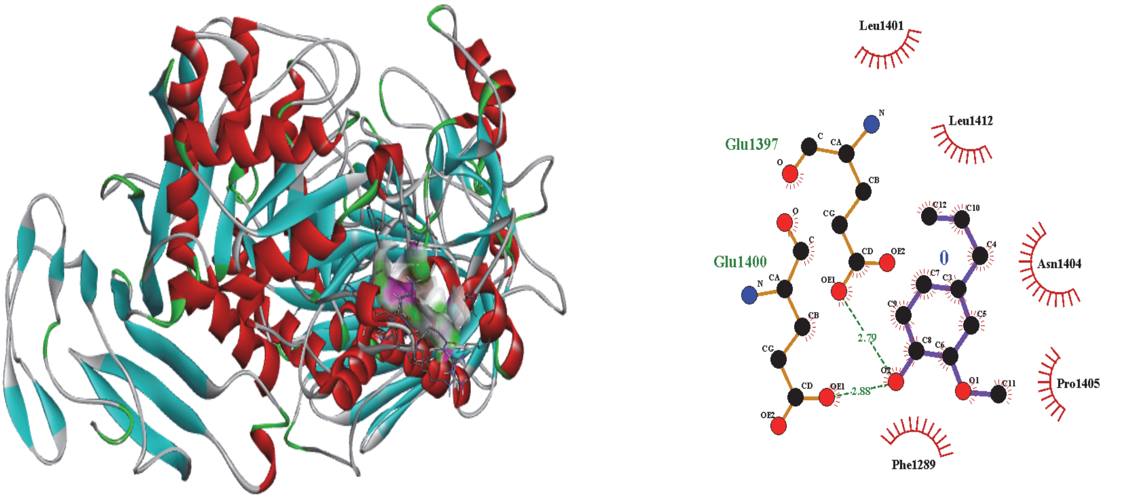

12

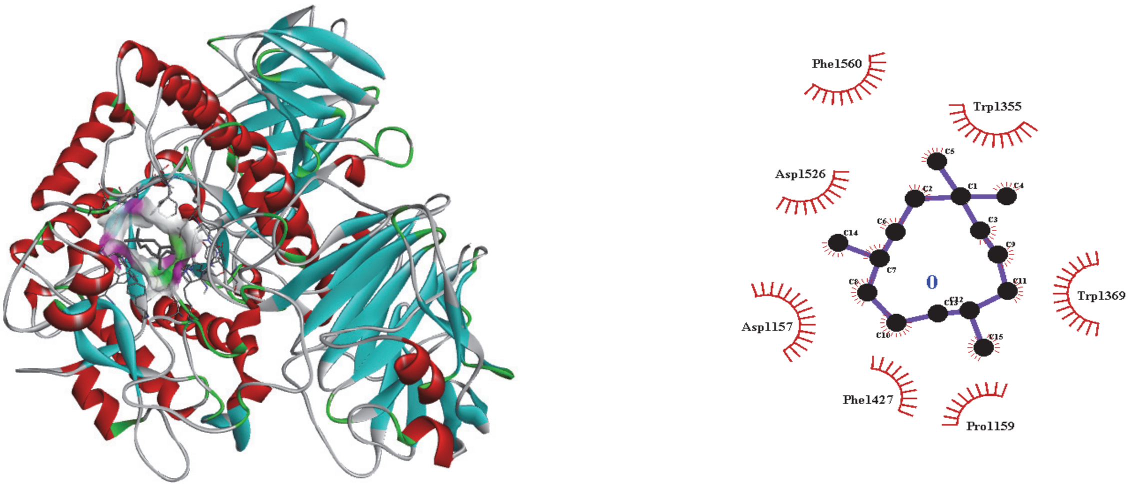

Figure S6. 3D H-bonding interactions of eugenol pose no. 2 [11] and α-humulene pose no. 1 [12] with binding sites of transcriptional regulator **3TOP**.

13

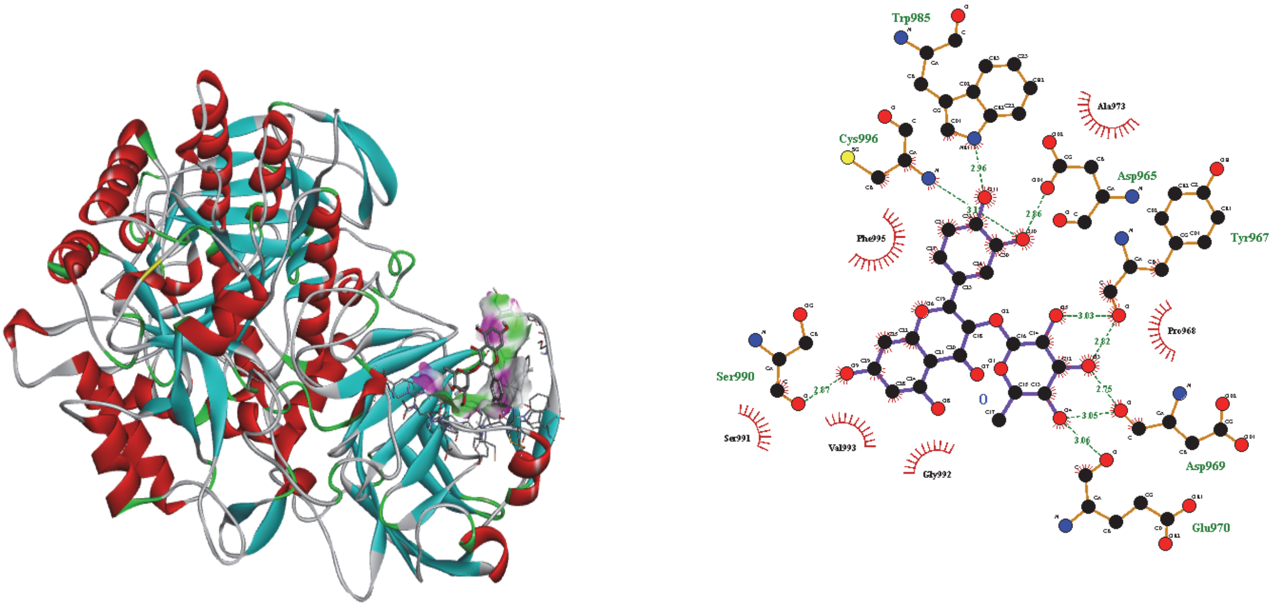

14

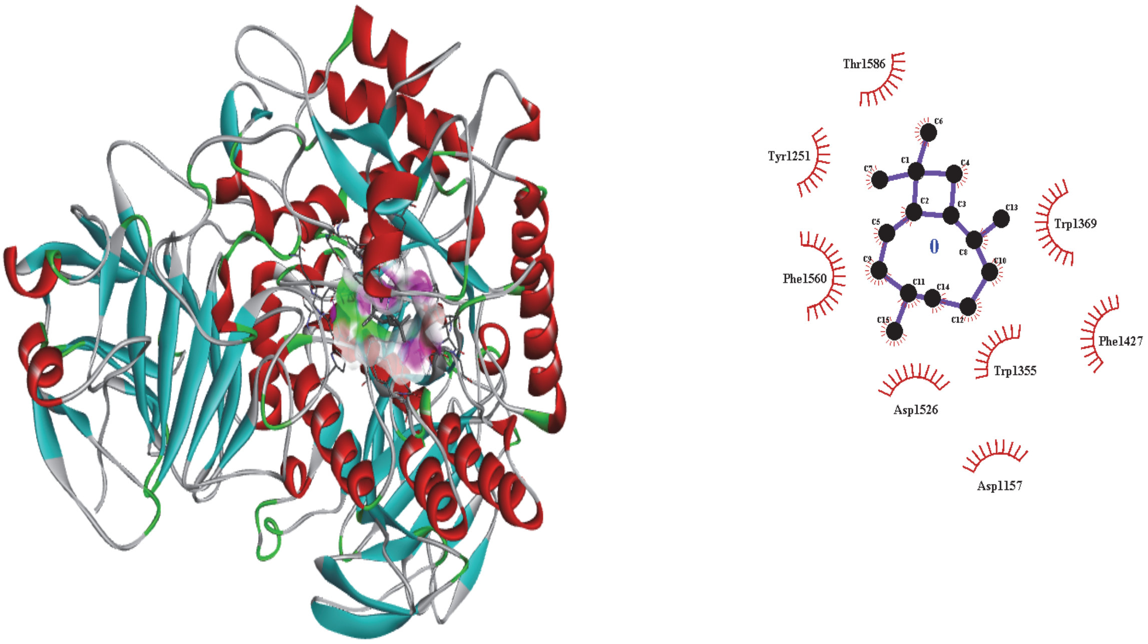

Figure S6. 3D H-bonding interactions of quercitrin pose no. 4 [13] and transcaryphyllene pose no. 1 [14] with binding sites of transcriptional regulator 3TOP.

15

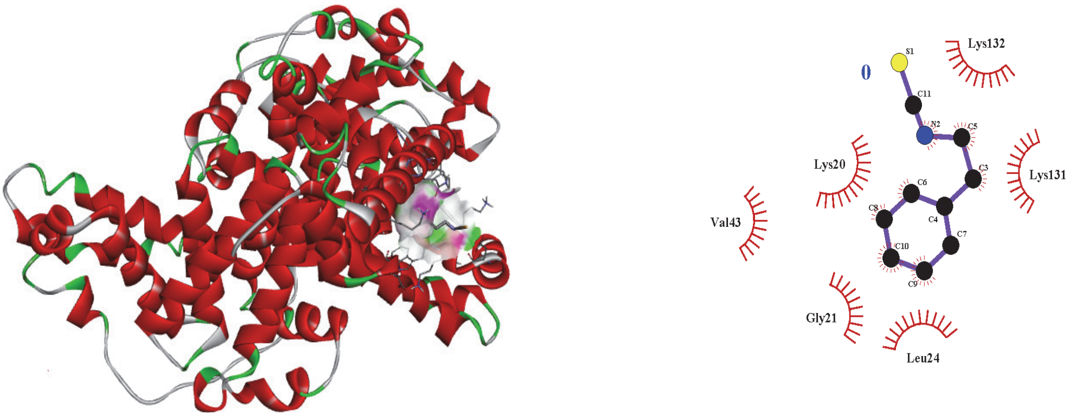

16

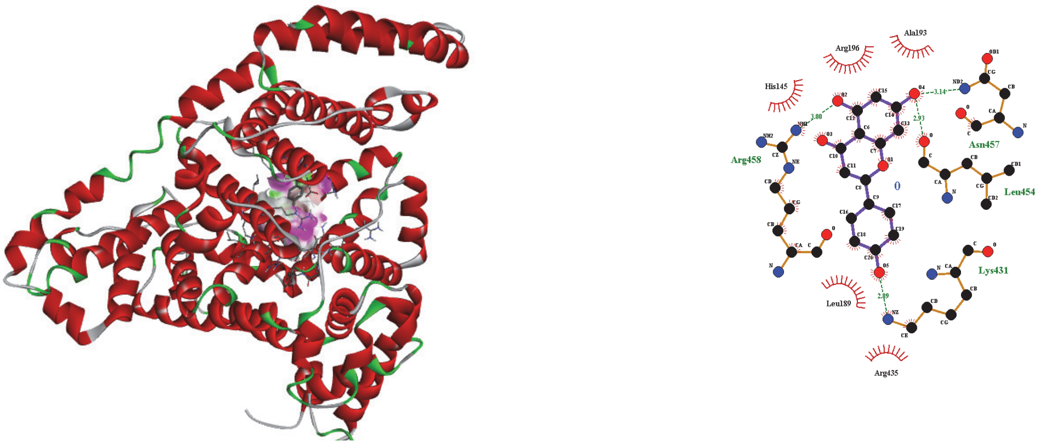

17

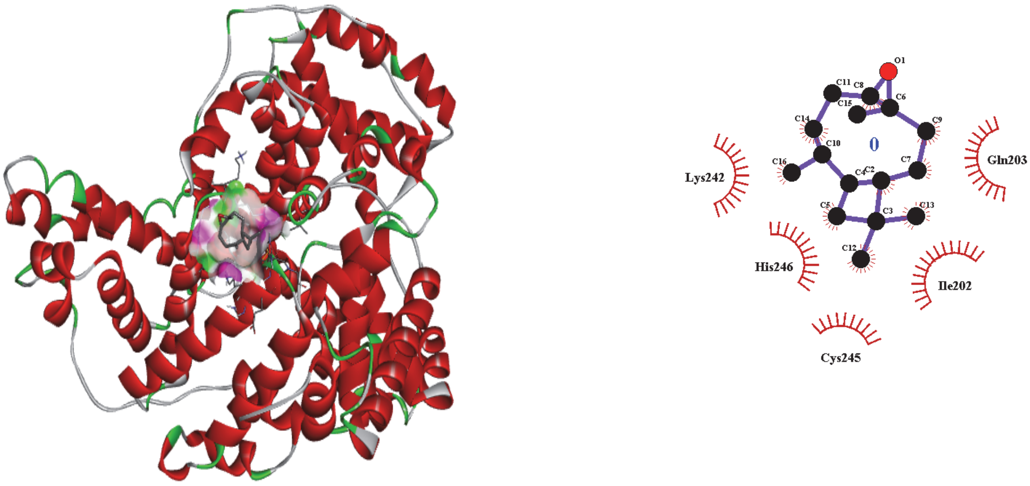

Figure S7. 3D H-bonding interactions of 2-phenylisothiocyanate pose no. 1 [15], apigenin pose no. 3[16] caryophyllene oxide pose no. 1 [17] with binding sites of transcriptional regulator **4F5S.**

18

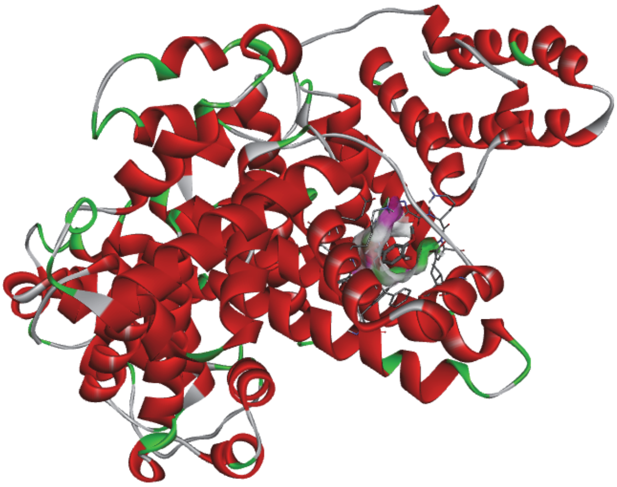

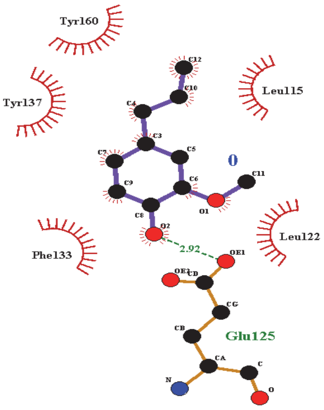

19

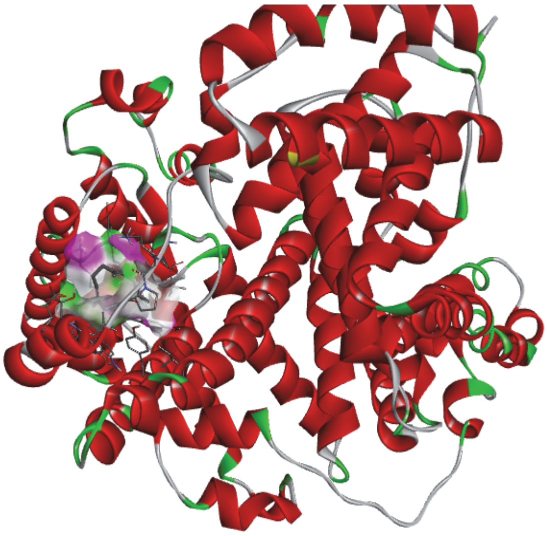

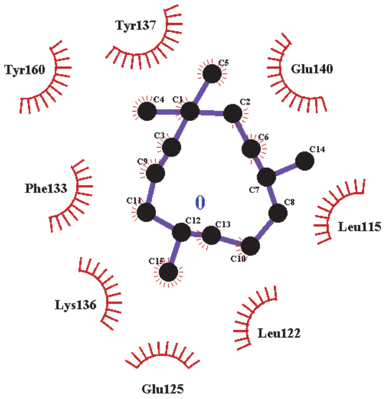

Figure S8. 3D H-bonding interactions of eugenol pose no. 1 [18], α-humulene pose no. 1[19] with binding sites of transcriptional regulator **4F5S.**

20

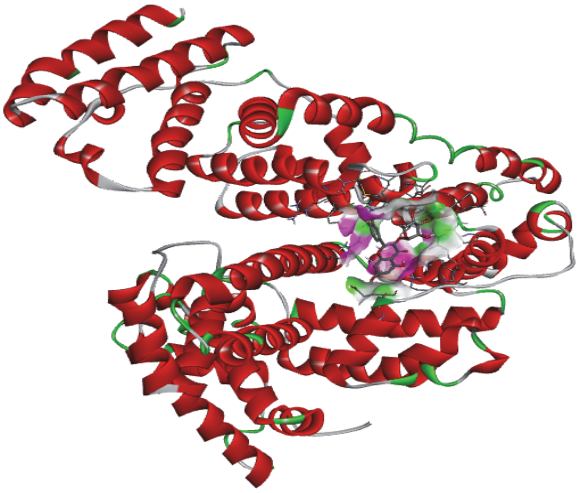

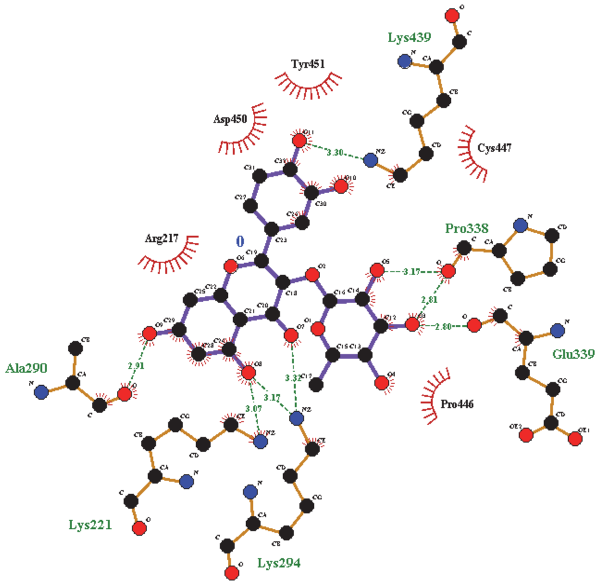

21

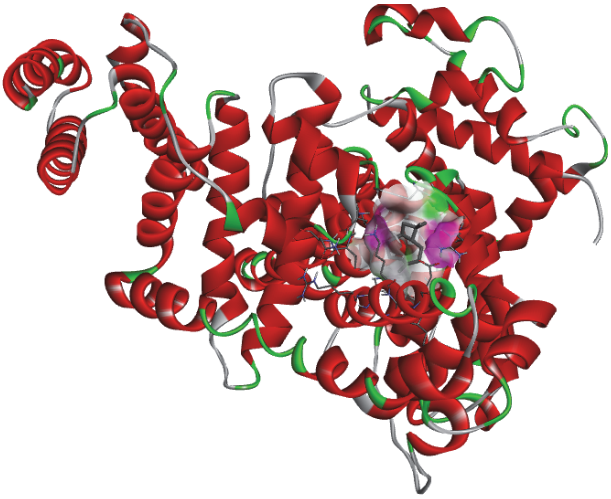

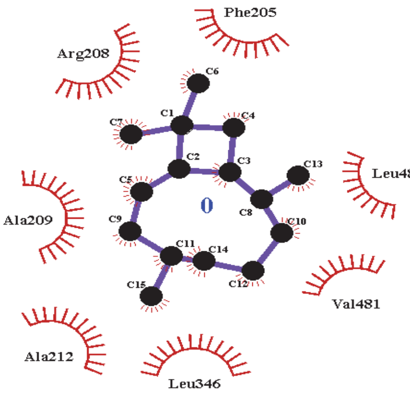

Figure S9. 3D H-bonding interactions of quercitrin pose no. 5 [20] and transcaryphyllene pose no. 1 [21] with binding sites of transcriptional regulator **4F5S**.
